# Transcriptome Analysis Reveals the Molecular Mechanism of *Potentilla anserina* L. Polysaccharides in Mitigating Zearalenone-Induced Oxidative Stress in Porcine Sertoli Cells

**DOI:** 10.3390/antiox14040439

**Published:** 2025-04-05

**Authors:** Haixia Shi, Zunqiang Yan, Hong Du, Shuangbao Gun

**Affiliations:** 1 College of Animal Science and Technology, Gansu Agricultural University, Lanzhou 730030, China; 2 Gansu Innovation Research Center for Swine Production Engineering and Technology, Lanzhou 730070, China

**Keywords:** transcriptome, *Potentilla anserina* L. polysaccharide, zearalenone, molecular mechanism

## Abstract

Zearalenone (ZEA) is a widespread mycotoxin that contaminates cereals and other animal feeds. Sertoli cells (SCs) are the main target of attack by many environmental toxins. Our previous study found that *Potentilla anserina* L. polysaccharides (PAP-1b) exhibited protective effects against ZEA-induced oxidative damage in testicular SCs. However, the regulatory mechanisms remain incompletely characterized. In this study, SCs were treated with a complete medium (CON group) or medium containing 150 μg/mL PAP-1b (PAP-1b group). After 4 h, 100 μM ZEA was added to the ZEA group and PAP-1b-ZEA group, respectively. Samples were collected after the cells continued to be incubated for 48 h and subsequently subjected to transcriptome sequencing. The results showed that 1018, 7183, and 1023 differentially expressed genes (DEGs) were screened in the CON-vs.-PAP-1b, CON-vs.-ZEA, and ZEA-vs.-PAP-1b-ZEA groups, respectively. Among them, glutathione peroxidase 1 (*GPX1*) emerges as a key gene within this antioxidant defense mechanism. In addition, these DEGs were significantly enriched in Gene Ontology (GO) terms related to oxidative stress as well as in MAPK and PI3K-AKT signaling pathways, suggesting that PAP-1b effectively mitigated ZEA-induced oxidative damage in SCs by regulating these signaling pathways. These results provide an essential basis for the further elucidation of the role of PAP-1b in mitigating ZEA-induced oxidative damage in SCs.

## 1. Introduction

Zearalenone (ZEA) is a mycotoxin with an estrogen-like structure generated by *Fusarium* species [1,2]. ZEA, a prevalent mycotoxin contaminant in global food chains, has stable chemical properties and is resistant to high temperatures, making it difficult to break down during processing [3,4]. It has been shown to possess immunotoxicity [5], genotoxicity [6], hepatotoxicity [7], nephrotoxicity [8], and reproductive toxicity [7]. Among these, reproductive toxicity is one of the most serious toxic effects of ZEA. This toxicity is mainly due to the high similarity between the chemical structure of ZEA and that of 17β-estradiol, an endogenous estrogen in animals, which enables it to competitively bind to the estrogen receptor and interfere with the normal hormone signaling pathway [9,10,11,12]. The International Agency for Research on Cancer (IARC) classified ZEA as a Group 3 carcinogen [13]. Beyond its endocrine-disrupting properties, ZEA mediates endoplasmic reticulum stress [14], DNA damage [15], and oxidative stress [16] through the production of reactive oxygen species (ROS) as an upstream control signal, thus leading to apoptosis [17]. Comparative metabolic studies reveal a descending toxicity gradient among its derivatives: α-ZAL > α-ZOL > β-ZAL > ZEA > β-ZOL. Notably, porcine models show heightened sensitivity attributed to preferential α-ZOL accumulation [18].

Currently, mycotoxin control in livestock feed primarily depends on physical, biological, and chemical methods. However, these methods have numerous limitations, such as high equipment costs, losses of essential nutrients, chemical residue, and decreased palatability. Recent studies have shown that natural ingredients with antioxidant activity extracted from plants can be effective in reducing ZEA-induced toxicity damage. For example, Lin et al. [19] revealed that betulinic acid was effective in ameliorating ZEA-induced oxidative stress and testicular dysfunction in mice by inhibiting the p38/ERK MAPK pathway and activating the Nrf2-mediated antioxidant defense system. Long et al. [20] showed that procyanidins were effective in ZEA-induced apoptosis in mouse Sertoli cells (SCs) via the Nrf2/ARE signaling pathway. In addition, Cao et al. [17] also demonstrated that lycopene was able to reduce the oxidative stress induced by ZEA in piglet SCs by activating the Nrf2 signaling pathway. Furthermore, *Potentilla anserina* L. is an edible and medicinal plant that grows mainly in the alpine regions of China [21]. *Potentilla anserina* L. contains various bioactive constituents including polysaccharides, phenols, flavonoids, terpenoids, etc., with more than 30% polysaccharides [22,23]. Previous studies have revealed that *Potentilla anserina* L. polysaccharides (PAP-1b) exhibit a variety of potential bioactivities and pharmacological effects such as antioxidant, anti-hypoxia, and immunomodulation properties [22,23,24]. However, the molecular regulatory mechanism regarding how PAP-1b attenuated ZEA-induced cytotoxic injury in porcine SCs has not been reported.

As the main gonadal organ of the male, the testis plays an important role in maintaining spermatogenesis and regulating androgen biosynthesis [25]. The testes mainly consist of germ cells, SCs, Leydig cells, and peritubular myoid cells. Among them, SCs exert a vital role in testicular development and spermatogenesis through contact with germ cells at all levels [26]. Increasing evidence suggests that SCs are prime targets for many environmental toxicants due to their unique biological properties and critical location in the reproductive system [27]. Therefore, SCs have become an ideal cell model to study the reproductive toxicity mechanism of environmental pollutants.

Transcriptome sequencing is a highly efficient and widely applied molecular biology analysis technique that provides a comprehensive and rapid analysis of the types and quantitative changes in all transcriptional products in an organism, thereby revealing the expression patterns of genes under specific conditions [28,29]. Recently, transcriptome sequencing technology has been widely used to reveal the regulatory mechanism of natural polysaccharide bioactivity. For example, Wang et al. [30] elucidated the immunomodulatory mechanisms of *Ganoderma atrium*-derived polysaccharides in activating myeloid-derived dendritic cells in murine models based on transcriptome sequencing. Shen et al. [31] investigated the cytoprotective mechanism of yam polysaccharide (CYP) in mitigating H_2_O_2_-triggered oxidative damage by transcriptome sequencing. However, to date, the molecular regulatory mechanism of PAP-1b to attenuate the cytotoxic damage of ZEA on porcine SCs using transcriptome sequencing technology has not been reported.

Our laboratory has confirmed that PAP-1b can effectively reduce the oxidative stress and apoptosis caused by ZEA on SCs [32]. However, its specific molecular regulatory mechanism remains unclear. Therefore, in this study, we used Illumina Novaseq 6000 platform (Illumina, Inc., San Diego, CA, USA) to conduct transcriptome sequencing and bioinformatics analysis to reveal the molecular regulatory mechanism of PAP-1b in alleviating ZEA-induced oxidative stress and toxicological damage in SCs. This work not only provides a new perspective for understanding the biological activity of PAP-1b but also provides a new strategy for solving the negative effects of ZEA on pig health and production performance.

## 2. Results

### 2.1. Data Filtering and Quality Assessment

Sequencing generated 567.27 M raw reads, with high-quality reads ranging from 46.04 to 47.42 M after quality control processing. Sequencing quality metrics demonstrated Q30 scores spanning 97.24–97.79%, coupled with a mean GC content of 51.27% (Supplementary Table S1). The reads after quality control were compared to the porcine reference genome, and the comparison rate ranged from 98.95% to 99.03%. The range of reads matched to a unique position in the reference genome sequence was 96.21% to 96.53%, while those reads matched to multiple positions in the reference genome sequence were 2.48% to 2.74% (Supplementary Table S2).

### 2.2. Analysis of Overall Gene Expression Levels

**Figure 1:**
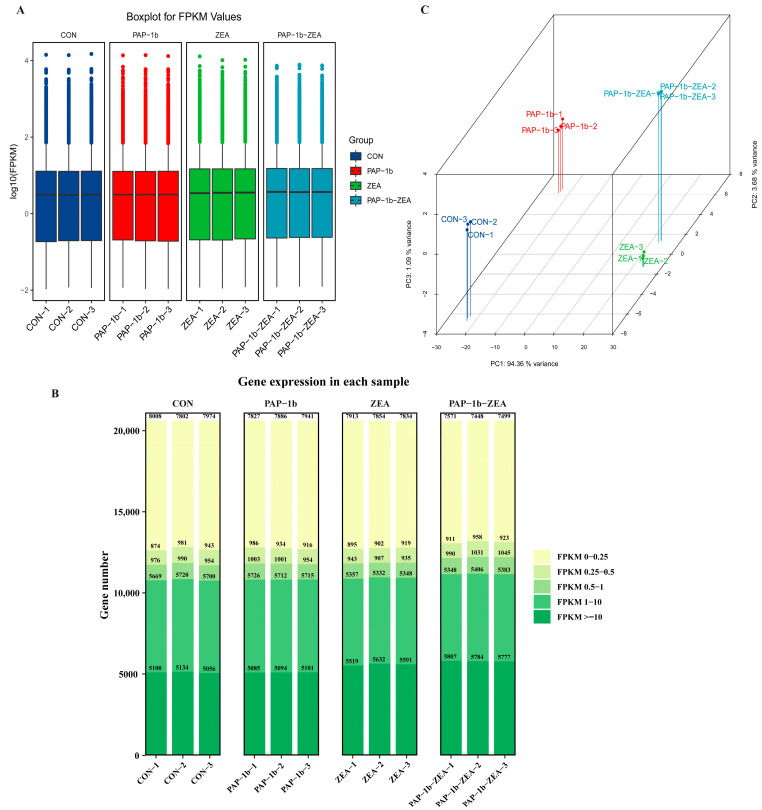
Analysis of overall gene expression level. (**A**) Boxplot of FPKM values for each sample gene; (**B**) distribution of FPKM expression for each sample; (**C**) PCA analysis plot for each sample.

As shown in Figure 1A, the maximum, third quartile, median, first quartile, and minimum values of gene expression of the 12 samples showed consistency. In addition, we divided the FPKM expression of genes into several intervals and counted the number of genes falling into these intervals in each sample (Figure 1B). The number of genes with FPKM values in the intervals of 0–0.25, 1–10, and greater than or equal to 10 was relatively higher, ranging from 5056 to 8008. In contrast, the number of genes with FPKM values in the two intervals of 0.25–0.5 and 0.5–1 was relatively smaller, ranging from about 874 to 1045, and this distributional feature revealed some differences in the number of gene expressions and the distribution of gene expression values in different samples. As shown in Figure 1C, the 12 samples were divided into four distinct fractions based on the principal component PCA1 (94.36%), principal component PCA2 (3.68%), and principal component PCA3 (1.09%). Three replicates within the group were clustered together, whereas the groups showed a clear distinction between them, thus suggesting that the samples within the groups were better replicated and the differences between the groups were larger. Collectively, the quality metrics demonstrate that the sequencing results are reliable.

### 2.3. Identification of DEGs

**Figure 2:**
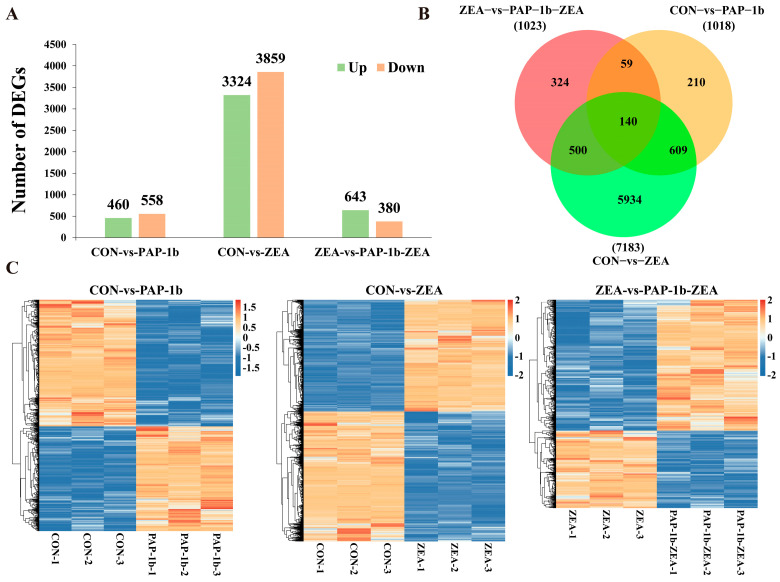
Analysis of DEGs. (**A**) Statistics of DEGs: CON-vs.-PAP-1b: 460 up-regulated and 558 down-regulated DEGs, CON-vs.-ZEA: 3324 up-regulated and 3859 down-regulated DEGs, and ZEA-vs.-PAP-1b-ZEA: 643 up-regulated and 380 down-regulated DEGs; (**B**) Venn diagram of DEGs for the three comparison groups; (**C**) clustering heatmap of DEGs.

As displayed in Figure 2A, compared to the CON group, 1018 gene changes (558 down- and 460 up-regulated) were observed in the PAP-1b group, whereas 7183 genes (3859 down- and 3324 up-regulated) were seen in the ZEA group. Additionally, a total of 1023 DEGs, including 380 down- and 643 up-regulated genes, were present in the PAP-1b-ZEA group compared to the ZEA group. Supplementary Tables S3–S5 listed more specific information regarding these DEGs. Venn diagram analysis revealed the co-expression of 140 DEGs between the three two-by-two comparison groups (Figure 2B). The clustering heatmap showed that there were significant differences in gene expression between the CON, PAP-1b, ZEA, and PAP-1b-ZEA groups, with obvious up- and down-regulation trends, suggesting that the mRNA expression pattern of the SCs was significantly affected by PAP-1b and ZEA (Figure 2C).

### 2.4. GO Function Annotation

**Figure 3:**
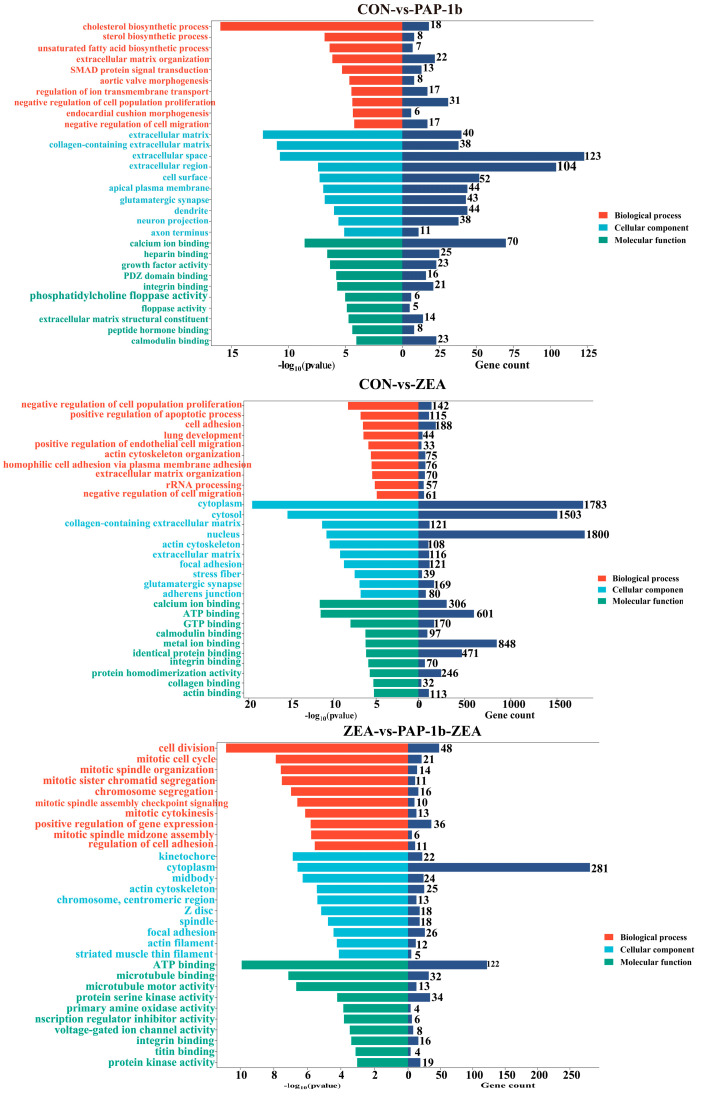
GO functional annotation of the DEGs. The left side of the X-axis represents −log10 (*p*-value), while the right side represents the number of genes under each GO term. The Y-axis lists GO terms, where red bars indicate biological processes, blue bars indicate cellular components and green bars indicate molecular functions.

GO functional annotation showed that 600 GO terms were significantly enriched in the CON-vs.-PAP-1b. Among them, 404 were related to biological processes, such as the regulation of ion transmembrane transport, extracellular matrix organization, and SMAD protein signal transduction; 87 were associated with cellular components, including extracellular matrix, collagen-containing extracellular matrix, and cell surface; and 109 were linked to molecular functions, such as growth factor activity, calcium ion binding, and peptide hormone binding. A total of 904 GO terms were significantly enriched in CON-vs.-ZEA. Among them, 572 were related to biological processes, such as the positive regulation of the apoptotic process, the negative regulation of cell population proliferation, and the negative regulation of cell migration; 161 were associated with cellular components, including cytosol, actin cytoskeleton, and focal adhesion; and 171 were linked to molecular functions, such as calcium ion binding, ATP binding, and actin binding. In ZEA-vs.-PAP-1b-ZEA, 525 GO terms were significantly enriched. Among them, 353 were related to biological processes, such as cell division, the mitotic cell cycle, and the regulation of cell adhesion; 84 were associated with cellular components, including cytoplasm, actin cytoskeleton, and actin filaments; and 88 were linked to molecular functions, such as ATP binding, transcription regulator inhibitor activity, and primary amine oxidase activity (Supplementary Table S6 and Figure 3).

### 2.5. KEGG Pathway Enrichment Analysis

**Figure 4:**
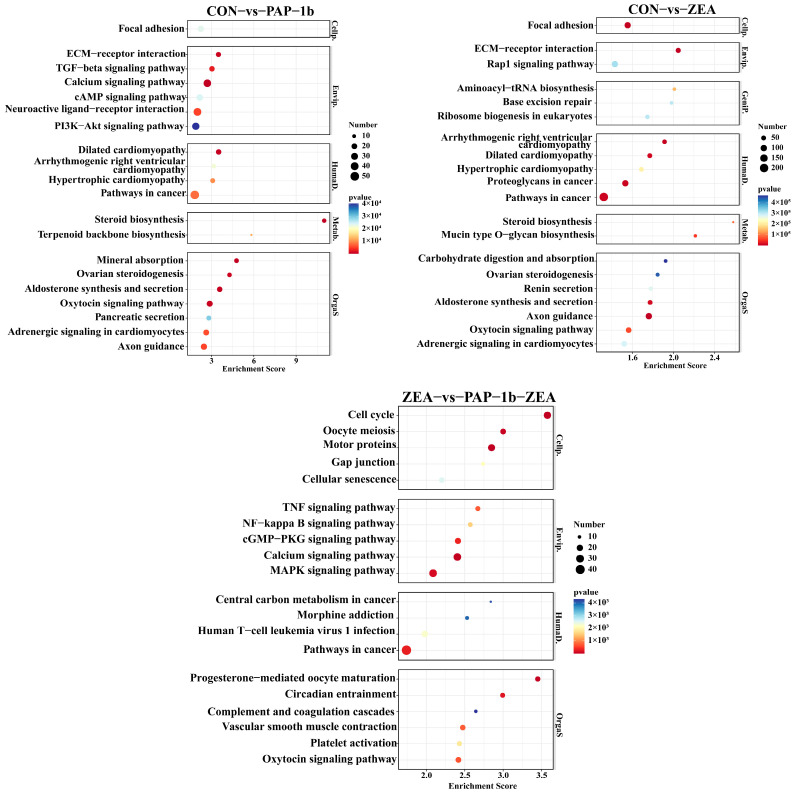
KEGG pathway enrichment of the DEGs.

KEGG pathway enrichment analysis indicated that DEGs in the CON-vs.-PAP-1b were mainly focused on focal adhesion, calcium signaling pathway, ECM-receptor interaction, PI3K-Akt signaling pathway, and other biological pathways; the DEGs in the CON-vs.-ZEA were mainly enriched in ECM-receptor interaction, focal adhesion, pathways in cancer, steroid biosynthesis, aminoacyl-tRNA biosynthesis, base excision repair, and ribosome biogenesis in eukaryotes; the DEGs in the ZEA-vs.-PAP-1b-ZEA were mainly focused on the MAPK signaling pathway, cell cycle, calcium signaling pathway, pathways in cancer, and complement and coagulation cascades (Supplementary Table S7 and Figure 4).

### 2.6. Further Screening of Key DEGs

**Figure 5:**
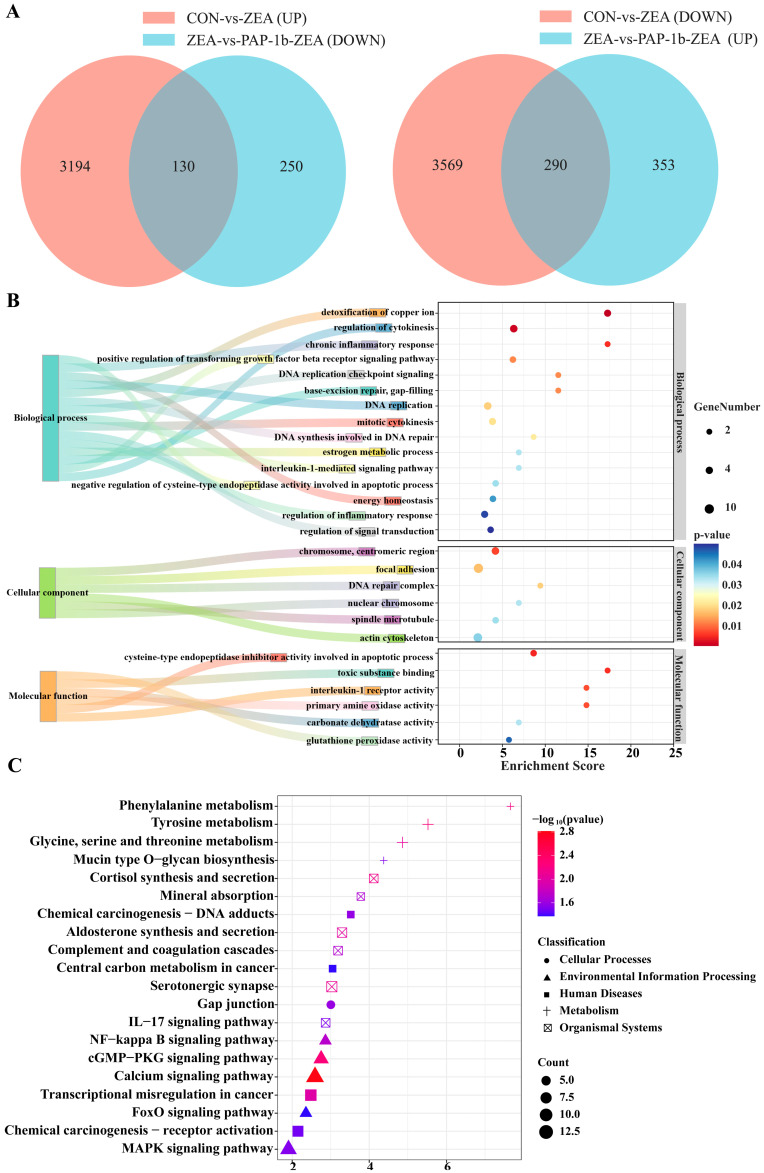
Key DEGs screening and functional enrichment analysis. (**A**) Venn diagram of key DEGs; (**B**) GO functional annotation; and (**C**) KEGG signaling pathway enrichment analysis.

To further screen the key DEGs for PAP-1b to alleviate ZEA-induced cellular damage, we focused on those DEGs that exhibited significant changes in expression levels in the CON-vs.-ZEA group, and these changes exhibited an opposite trend in the ZEA-vs.-PAP-1b-ZEA group. The Venn diagram shows that the expression pattern of 420 DEGs was reversed after pretreatment with PAP-1b, of which 130 genes up-regulated by ZEA treatment were down-regulated by PAP-1b interference, while 290 genes down-regulated by ZEA treatment were up-regulated (Supplementary Table S8 and Figure 5A).

GO analysis results displayed that 420 DEGs were enriched in 243 GO terms. These terms were related to biological processes such as DNA replication checkpoint signaling, the detoxification of copper ions, DNA replication, DNA synthesis involved in DNA repair, and the regulation of inflammatory response. Regarding cellular components, these DEGs were closely related to structures such as focal adhesion, DNA complex repair, nuclear chromosomes, and actin cytoskeleton. In addition, the molecular functions exhibited by these DEGs mainly included interleukin-1 receptor activity, primary amine oxidase activity, and glutathione peroxidase activity (Supplementary Table S9 and Figure 5B).

These DEGs were mainly enriched in the Gap junction, NF-kappa B signaling pathway, MAPK signaling pathway, FoxO signaling pathway, complement and coagulation cascades, transcriptional misregulation in cancer, IL-17 signaling pathway, and other key signaling pathways (Supplementary Table S10 and Figure 5C).

### 2.7. PPI Network

**Figure 6:**
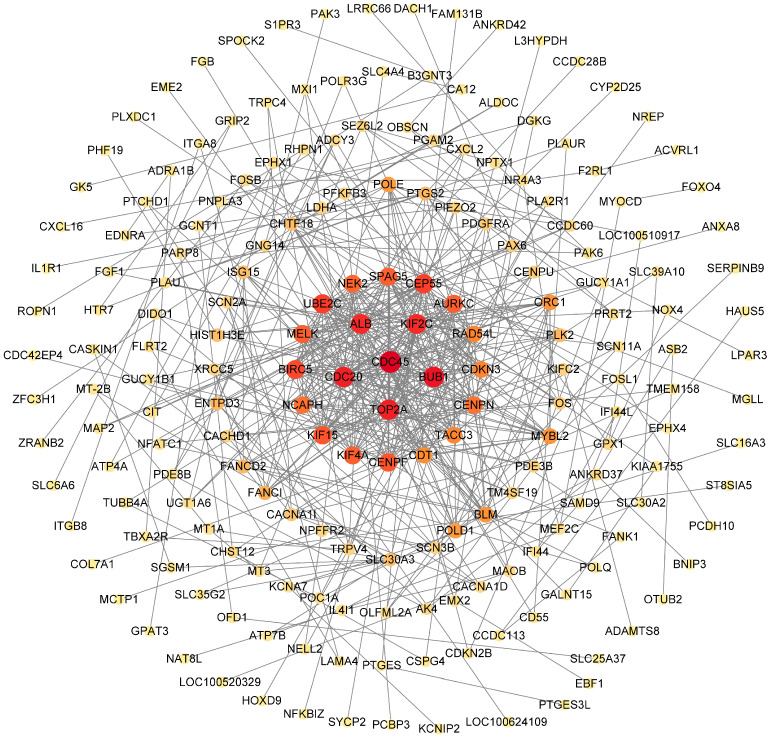
PPI interaction network of key proteins. The shade of the color reflects the degree of protein connectivity, with darker colors indicating a higher degree of connectivity. The degree of connectivity for the first circle is 30, the second circle ranges from 25 to 29, the third circle ranges from 15 to 24, the fourth circle ranges from 5 to 14, the fifth circle ranges from 3 to 4, the sixth circle is 2, and the seventh circle is 1.

The PPI network contained 196 nodes, which were tightly connected through 500 edges and interwoven into an intricate network of interactions (Figure 6). We screened 22 core proteins: CDC45, BUB1, CDC20, TOP2A, KIF2C, ALB, CEP55, UBE2C, BIRC5, CENPF, KIF15, KIF4A, SPAG5, MELK, AURKC, NEK2, NCAPH, CENPN, RAD54L, TACC3, CDKN3, and CDT1. These proteins were located at the center of the PPI network with high connectivity, suggesting that they play crucial roles in the protein interactions network. In addition, these core proteins were closely related to key biological processes such as cell cycle regulation, mitotic progression, and DNA replication and repair.

### 2.8. Validation of Sequencing Results

**Figure 7:**
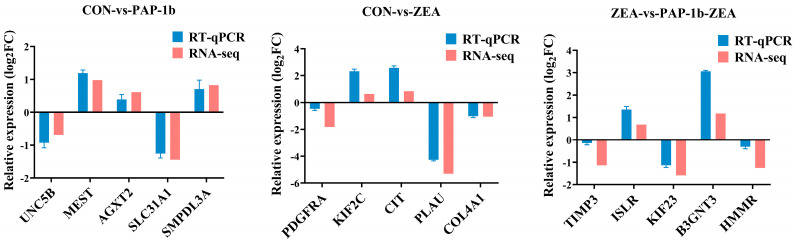
RT-qPCR validation of DEGs.

To validate the accuracy of the transcriptome sequencing data, we randomly selected 15 DEGs, including *UNC5B*, *MEST*, *AGXT2*, *SLC31A1*, *SMPDL3A*, *PDGFRA*, *KIF2C*, *CIT*, *PLAU*, *COL4A1*, *TIMP3*, *ISLR*, *KIF23*, *B3GNT3*, and *HMMR* to perform RT-qPCR. The results showed that, in CON-vs.-PAP-1b, *UNC5B* and *SLC31A1* exhibited a down-regulation trend, while *MEST*, *AGXT2*, and *SMPDL3A* displayed an up-regulation trend, which was consistent with the transcriptome sequencing data; in CON-vs.-ZEA, *PDGFRA*, *PLAU*, and *COL4A1* exhibited a down-regulation trend, while *KIF2C* and *CIT* exhibited an up-regulation trend, which was consistent with the transcriptome sequencing data; and in ZEA-vs.-PAP-1b-ZEA, *TIMP3*, *KIF23*, and *HMMR* were down-regulated, while *ISLR* and *B3GNT3* were up-regulated, which was consistent with the transcriptome sequencing data (Figure 7). These results indicated that the sequencing data in this study were reliable.

## 3. Discussion

Transcriptome sequencing has emerged as an efficient and widely used technique to study the molecular mechanisms linked to the pharmacological effects of natural polysaccharides [33]. The results of the previous experiments of our group revealed that PAP-1b was able to alleviate ZEA-trigged oxidative damage and cytotoxicity in porcine SCs. However, the specific molecular regulatory mechanisms are unknown. To elucidate the potential mechanism of the mitigating effect of PAP-1b on ZEA-induced oxidative stress and cytotoxicity in SCs, the present study, for the first time, screened the key candidate genes of PAP-1b for mitigating ZEA-induced Sertoli cells toxicity using transcriptome sequencing technology and functionally enriched these genes by bioinformatics analysis.

Transcriptome sequencing analysis indicated that 1018 genes were significantly changed in the PAP-1b group compared with the CON group. GO functional annotation suggested that these DEGs were mainly enriched in the biological process, such as the sterol biosynthetic process, cholesterol biosynthetic process, and unsaturated fatty acid biosynthetic process. Cholesterol and sterols are essential for the composition of cell membranes and the synthesis of reproductive hormones [34], whereas unsaturated fatty acids have an indispensable role in maintaining the fluidity and stability of cell membranes [35]. Thus, these biological processes are essential for maintaining the normal physiological and reproductive function of testicular SCs. In addition, a series of GO terms closely related to oxidative stress, inflammatory response, and lipid metabolism were significantly enriched, including the lipoxygenase pathway, arachidonic acid metabolic process, and peroxidase activity. Notably, all of these GO terms involve glutathione peroxidase 1 (*GPX1*), an important antioxidant enzyme that plays critical roles in maintaining cellular redox homeostasis, protecting cells from oxidative stress, and regulating diverse physiological and pathological processes [36,37].

Compared with the CON group, 7183 genes were significantly changed in the ZEA-treated group, of which 3859 were down-regulated and 3324 were up-regulated, suggesting that ZEA treatment induced large-scale transcriptional reprogramming of the SCs. GO functional annotation revealed that the top two biological processes in which these DEGs were significantly enriched were negative regulation of cell population proliferation and positive regulation of the apoptotic process, suggesting that ZEA may exacerbate the SCs damage through the dual mechanism of inhibiting cell proliferation and promoting apoptosis. In addition, these DEGs were also significantly enriched with GO terms related to oxidative stress, including cellular response to oxidative stress, response to redox state, glutathione metabolic process, response to oxidative stress, etc. Genes associated with these GO terms include *G6PD*, *GSR*, *NQO1*, *PRDX2*, *SELENON*, *SELENOS*, *SESN2*, *SOD2*, *SLC7A11*, *GSTA1*, *GSTK1*, *GSTM3*, *GSTZ1*, *CYGB*, *GPX1*, *GPX3*, *GPX8*, *GSS*, *OXR1*, *SRXN1*, and *TXNRD1*, which play crucial roles in the oxidative stress process by encoding various antioxidant enzymes and proteins. For example, G6PD is a key enzyme of the pentose phosphate pathway, which is essential for maintaining intracellular NADPH levels and the reduced state of glutathione [38]; GSR catalyzes the reduction of oxidized glutathione to reduced glutathione, which protects cells from oxidative damage [39]; and NQO1, PRDX2, and SOD2 play antioxidant roles by either directly scavenging ROS or regulating intracellular oxidative reduction balance to exert antioxidant effects [40,41]. In addition, GST family members (e.g., GSTA1, GSTK1, GSTM3, and GSTZ1) are involved in glutathione-binding reactions that help cells detoxify exogenous substances and oxidative products [42], whereas GPX family members (e.g., GPX1, GPX3, and GPX8) catalyze the reduction of hydrogen peroxide and lipid peroxides, which further protects the cells from damage caused by oxidative stress [43]. In the CON-vs.-ZEA group of this study, *G6PD*, *PRDX2*, *SELENON*, *SELENOS*, *CYGB*, and *TXNRD1* were down-regulated, while *GSR*, *NQO1*, *SOD2*, *GSTA1*, *GSTK1*, *GSTM3*, *GSTZ1*, *GPX1*, *SESN2*, *SLC7A11*, *GSS*, *OXR1*, and *SRXN1* were up-regulated, and these genes modulated each other to regulate the oxidative and antioxidant status of SCs.

Studies have indicated that the MAPK signaling pathway is closely related to oxidative stress and plays a crucial role in cellular biological signaling [44,45]. The PI3K-Akt signaling pathway is a key signaling network in cells, which is involved in a variety of physiological processes such as cell survival, metabolism, and apoptosis [46]. In addition, the glutathione metabolic pathway has an essential role in maintaining cellular redox homeostasis and protecting cells from oxidative stress. Glutathione (GSH), a tripeptide composed of glutamate, cysteine, and glycine, is the main endogenous antioxidant in cells, and its main function is to scavenge ROS and other free radicals, thereby preventing oxidative damage to cellular components such as proteins, lipids, and DNA [47,48]. The KEGG results of the present study revealed that DEGs in the CON-vs.-ZEA group were significantly enriched in the MAPK signaling pathway, the PI3K-Akt signaling pathway, and the glutathione metabolic pathway, suggesting that ZEA-induced oxidative stress in SCs may be closely related to these pathways.

Compared with ZEA, 1023 genes were significantly altered in the PAP-1b-ZEA-treated group. It is noteworthy that *GPX1* also exhibited differential expression, but its expression trend was down-regulated, which was opposite to that of *GPX1* expression in the CON-vs.-ZEA group. This phenomenon suggests that *GPX1* may play different roles under different treatment conditions. In the CON-vs.-ZEA group, the up-regulated expression of *GPX1* might be to protect against ZEA-induced oxidative damage. However, in the ZEA-vs.-PAP-1b-ZEA group, the expression trend of *GPX1* was reversed and showed a down-regulation trend, which may mean that the ZEA-induced oxidative stress response in SCs was attenuated under pretreatment with PAP-1b, or that there were other antioxidant mechanisms at play, which reduced the dependence of *GPX1*. In summary, the expression changes in GPX1 reflect the complex regulatory mechanisms displayed by cells in response to different external stimuli.

Numerous studies have revealed that plant polysaccharides can exert antioxidant effects by modulating PI3K-Akt signaling pathways and MAPK signaling pathways. For example, Chen et al. [46] suggested that Cyclocarya paliurus polysaccharide protected L02 cells from H_2_O_2_-induced oxidative damage by regulating mitochondrial function, oxidative stress, and PI3K-Akt and MAPK signaling pathways. Similar research results appeared in the study of Liu et al. [49], who found that selenizing astragalus polysaccharide inhibited the autophagy process by activating the PI3K-Akt pathway, which effectively attenuated PCV2 replication promotion caused by oxidative stress. In the present study, we also obtained similar results that DEGs in the ZEA-vs.-PAP-1b-ZEA group were significantly enriched in the PI3K-Akt and MAPK signaling pathways. This finding suggests that the role of PAP-1b in alleviating ZEA-induced oxidative stress in SCs may be closely related to these signaling pathways.

Although this study has advanced our understanding of the molecular mechanisms by which PAP-1b alleviated ZEA-induced oxidative stress, several critical scientific questions remain to be addressed. For instance, the regulatory role of *GPX1* in PAP-1b-mediated protection against ZEA-induced oxidative stress through gene knockdown or overexpression experiments. Additionally, constructing ZEA-exposed porcine models is essential to validate the in vivo efficacy of PAP-1b in mitigating ZEA-induced reproductive toxicity. These investigations will provide a theoretical foundation for developing functional premixes containing PAP-1b as feed additives.

## 4. Materials and Methods

### 4.1. PAP-1b Preparation and SCs Culture

All animal experimentation protocols were conducted under the Experimental Animal Ethics Committee of Gansu Agricultural University, ensuring that the experimental processes adhere to ethical principles (Approval No. GAU-LC-2018-054). PAP-1b was extracted and purified by our laboratory previously from *Potentilla anserina* L. roots. Landrace boars’ primary SCs were isolated and cultured in our laboratory. The procedure was as follows: testes (*n* = 10, bilateral) were collected from five 21-day-old healthy Landrace boars. After removing the tunica albuginea and epididymis, the testicular tissues were minced and digested with 1 mg/mL collagenase IV for 50 min. Subsequently, 0.25% trypsin was added, and the digestion was continued in a 37 °C water bath for 20 min. The reaction was terminated with a complete medium containing 15% fetal bovine serum (FBS), 84% high-glucose DMEM, and 1% penicillin–streptomycin. The digested suspension was sequentially filtered through 100 μm, 70 μm, and 40 μm cell strainers. After centrifugation, cells were seeded into culture flasks and incubated at 37 °C with 5% CO₂. Six hours later, the medium was replaced with fresh medium to remove non-adherent germ cells. Upon reaching 80% confluence, cells were treated with 0.05% trypsin and re-plated. This purification step was repeated 1–2 times to obtain purified SCs.

### 4.2. Experimental Design

SCs with good growth conditions were seeded at a density of 2 × 10^5^ cells/mL. After the SCs were cultured into dense monolayers, they were divided into four groups, i.e., CON group, PAP-1b group, ZEA group, and PAP-1b-ZEA group. Three replicates were set up in each group, and each replicate had approximately 3 × 10⁶ SCs. CON group: medium only; PAP-1b group: medium containing 150 μg/mL PAP-1b (the optimal concentration of PAP-1b to alleviate ZEA-induced oxidative stress identified in the previous stage); ZEA group: medium containing 100 μM ZEA (the concentration used for constructing the oxidative stress model that was determined in the previous stage); PAP-1b-ZEA group: the cells were pretreated with 150 μg/mL PAP-1b medium for 4 h, and then 100 μM ZEA was added. According to the optimal conditions as determined previously, the four groups of SCs were incubated for 48 h.

### 4.3. RNA Extraction and Quality Assessment

Total RNA was extracted from each SC sample (approximately 3.5 × 10⁶ cells) using Trizol reagent (Invitrogen, Carlsbad, CA, USA). The concentration and purity were spectrophotometrically assessed (NanoDrop 2000, Wilmington, DE, USA) through absorbance ratios at 260/280 nm. Subsequently, we assessed RNA integrity using an Agilent 2100 Bioanalyzer (Agilent Technologies, Santa Clara, CA, USA), indicated by RNA integrity number (RIN). The library construction requirements were met when the total RNA concentration was ≥100 ng/μL, the OD 260/280 value was between 1.8 and 2.2, the RIN ≥ 7, and 28S/18S ≥ 0.7. The results of total RNA quality testing in this study are shown in Supplementary Table S11.

### 4.4. Library Construction and Sequencing

Poly(A) mRNA was enriched from the total RNA of SCs using magnetic beads with oligo (dT), and these mRNAs were used as templates to synthesize first-strand cDNAs using six-base random primers. Next, second-strand cDNAs were synthesized using DNA polymerase I, RNase H, and dUTP solution. Subsequently, the QiaQuick PCR kit (Qiagen, Hilden, Germany) was used to purify and perform end repairs to these double-stranded cDNAs, add A tails, and ligate sequencing junctions. Fragment size selection and PCR amplification were then performed. Finally, sequencing was performed using the Illumina Novaseq 6000 sequencing platform at Shanghai Ouyi Biotech Company and 150 bp double-ended reads were generated. Raw data were uploaded to the NCBI Sequence Read Archive (SRA) database under the accession number PRJNA1158769.

### 4.5. Raw Data Quality Control and Sequence Comparison Analysis

We used fastp [50] software (version 0.20.0, Haplox, Shenzhen, China) to preprocess the original raw reads—including removing reads containing splice contamination, reads with low-quality bases, and reads containing undetermined bases—and finally, high-quality clean reads were used for subsequent analyses. Subsequently, high-quality reads were mapped to the Sus scrofa reference genome (Sscrofa11.1) via HISAT2 [51], enabling transcriptional locus annotation at the reference genome or gene.

### 4.6. Identification of Differentially Expressed Genes (DEGs)

Transcript quantification was performed through the alignment of the protein-coding region reads using HTSeq-count [52], with transcript abundance normalized via FPKM (Fragments Per Kilobase of exon model per Million mapped fragments) [53]. Differential expression analysis between different treatment groups was conducted in DESeq2 [54], applying stringent thresholds of fold change (FC) greater than 1.5 or less than 1/1.5 and q-value < 0.05 for DEG identification.

### 4.7. Functional Enrichment Analysis of DEGs

GO functional annotation of DEGs was performed using the Gene Ontology database (GO; https://www.geneontology.org/, accessed on 3 July 2024). Meanwhile, a Kyoto Encyclopedia of Genes and Genomes (KEGG) pathway enrichment analysis of DEGs was performed using the KEGG database (https://www.genome.jp/kegg/, accessed on 3 July 2024), and significance was determined by a hypergeometric test. When the *p*-value was less than 0.05, we considered the pathway significantly enriched. In addition, we constructed a protein–protein interaction network (PPI) by the String database (https://cn.string-db.org/, accessed on 3 July 2024) and optimized it using Cytoscope 3.9.0. The key proteins were further screened based on the strict screening criteria of node degree ≥ 15.

### 4.8. Quantitative Real-Time Reverse Transcription–Polymerase Chain Reaction (RT-qPCR)

Total RNA (1 μg) extracted from SCs was converted into cDNA using the Evo M-MLV RT Mix Kit with gDNA Clean for qPCR Ver. 2 (Accurate Biotechnology, Changsha, China). Subsequently, RT-qPCR amplification was performed on a Roche LightCycler 480 (Roche, Mannheim, Germany) with the following 20 μL reaction system: 10 μL 2× SYBR Green Pro Taq HS Pre-mix (Accurate Biotechnology, Changsha, China), 0.8 μL each of forward/reverse primers (10 μM), 2 μL cDNA, and 6.4 μL RNase-free H_2_O. Gene expression quantification utilized the 2^−ΔΔCt^ method normalized to *β-actin* [55], with primer sequences detailed in Supplementary Table S12.

### 4.9. Statistical Analysis

Data were analyzed with GraphPad Prism 9.0 (GraphPad Software, San Diego, CA, USA). Results are presented as means ± standard deviation (S.D.).

## 5. Conclusions

In conclusion, this study investigated for the first time the molecular regulatory mechanism of PAP-1b in alleviating ZEA-induced oxidative stress in SCs. The results demonstrated that PAP-1b effectively alleviated ZEA-induced oxidative stress in SCs by modulating key signaling pathways, including MAPK and PI3K-AKT, and regulating the expression of critical antioxidant enzymes such as *GPX1*. These findings not only enhance our understanding of ZEA toxicity but also suggest the potential of PAP-1b as a natural antioxidant or feed additive for mitigating ZEA-induced reproductive toxicity.

## Data Availability

The raw data used in this study have been uploaded to the NCBI Sequence Read Archive (SRA) database under the accession number: PRJNA1158769.

## Supplementary Materials

The following supporting information can be downloaded at https://www.mdpi.com/article/10.3390/antiox14040439/s1. Table S1: Data filtering statistics. Table S2: Mapping results with porcine reference genome. Table S3: List of differentially expressed mRNAs between CON and PAP-1b. Table S4: List of differentially expressed mRNAs between CON and ZEA. Table S5: List of differentially expressed mRNAs between ZEA and PAP-1b-ZEA. Table S6: GO annotation of differentially expressed mRNAs. Table S7: KEGG analysis of differentially expressed mRNAs. Table S8: List of key DEGs. Table S9: GO annotation of key DEGs. Table S10 KEGG analysis of key DEGs. Table S11 Total RNA quality assessment. Table S12: Primer sequence information.
